# Microbial surfactants: A journey from fundamentals to recent advances

**DOI:** 10.3389/fmicb.2022.982603

**Published:** 2022-08-04

**Authors:** Dimple S. Pardhi, Rakeshkumar R. Panchal, Vikram H. Raval, Rushikesh G. Joshi, Peter Poczai, Waleed H. Almalki, Kiransinh N. Rajput

**Affiliations:** ^1^Department of Microbiology and Biotechnology, University School of Sciences, Gujarat University, Ahmedabad, Gujarat, India; ^2^Department of Biochemistry and Forensic Science, University School of Sciences, Gujarat University, Ahmedabad, Gujarat, India; ^3^Finnish Museum of Natural History, University of Helsinki, Helsinki, Finland; ^4^Department of Pharmacology, College of Pharmacy, Umm Al-Qura University, Makkah, Saudi Arabia

**Keywords:** biodegradable, emulsification, *Pseudomonas* spp., rhamnolipid, surface tension, surfactin

## Abstract

Microbial surfactants are amphiphilic surface-active substances aid to reduce surface and interfacial tensions by accumulating between two fluid phases. They can be generically classified as low or high molecular weight biosurfactants based on their molecular weight, whilst overall chemical makeup determines whether they are neutral or anionic molecules. They demonstrate a variety of fundamental characteristics, including the lowering of surface tension, emulsification, adsorption, micelle formation, etc. Microbial genera like *Bacillus* spp., *Pseudomonas* spp*., Candida* spp., and *Pseudozyma* spp. are studied extensively for their production. The type of biosurfactant produced is reliant on the substrate utilized and the pathway pursued by the generating microorganisms. Some advantages of biosurfactants over synthetic surfactants comprise biodegradability, low toxicity, bioavailability, specificity of action, structural diversity, and effectiveness in harsh environments. Biosurfactants are physiologically crucial molecules for producing microorganisms which help the cells to grasp substrates in adverse conditions and also have antimicrobial, anti-adhesive, and antioxidant properties. Biosurfactants are in high demand as a potential product in industries like petroleum, cosmetics, detergents, agriculture, medicine, and food due to their beneficial properties. Biosurfactants are the significant natural biodegradable substances employed to replace the chemical surfactants on a global scale in order to make a cleaner and more sustainable environment.

## Introduction

Now-a-days, microbial surfactants are taking place in humans’ lifestyles abundantly, a lavish component of their routine products like cosmetics, food additives, and detergents. They are also widely used in the petroleum, medical, pharmaceutical, agricultural, and environmental sectors. Using biodegradable microbial surfactants instead of synthetic surfactants will help improve the economy and reduce environmental issues (Bhardwaj et al., 2013). The hazardous effluents produced during the manufacturing of synthetic surfactants have a negative impact on the environment. Hence, their market attractiveness has fallen despite their cost-effectiveness. Microbial surfactants are natural, biodegradable, and non-toxic, and as a result, their market demand is steadily increasing. The global market size of chemical surfactants is projected to reach a CAGR (compound annual growth rate) of 5.3% from 2020 to 2027 (Dixit et al., 2020), while for biosurfactants it is expected to grow over 5.5% CAGR between 2020 and 2026, especially for rhamnolipids it will possibly reach over USD 145 million (Ahuja and Singh, 2020).

The most extensively used microorganisms for biosurfactant production involve *Pseudomonas* spp. and *Bacillus* spp. from oil-contaminated sites, effluent, wastewater, etc. Besides these, some fungi like *Candida* spp., *Torulopsis* spp., *Pichia* spp., *Aspergillus* spp. (Bhardwaj et al., 2013) and marine microbes like *Alcanivorax borkumensis*, *Alcaligenes* spp., *Arthrobacter* spp., *Myroides* spp., *Yarrowia lipolytica*, *Pseudomonas nautical* (Maneerat, 2005) are also reported with a substantial amount of biosurfactants. The biosynthetic pathway for biosurfactant production in microorganisms depends on the substrates and the cultural conditions, making them assorted in chemical composition. Biosurfactants range from low molecular weight to high weight and comprise glycolipids, lipopeptides, neutral lipids, phospholipids, and polymeric biosurfactants (Shah et al., 2016).

The carbon source may come from hydrocarbons, carbohydrates, and lipids, which may be used separately or in combination. Various chromatographic and spectroscopic methods confirm these surface-active compounds’ chemical structure and functional groups. Biosurfactants can also be produced from cheap raw materials from large quantities of agricultural byproducts/waste (Bhardwaj et al., 2013). The process of economics and environmental credentials makes biosurfactants more attractive when produced using relatively simple and inexpensive waste products as substrates. The present review deals with fundamental aspects of microbial surfactants, including their classes, properties, producing microbes, biosynthesis, production, recovery, and characterization, along with the recent market potential, patents, and novel applications.

## Classification

**FIGURE 1:**
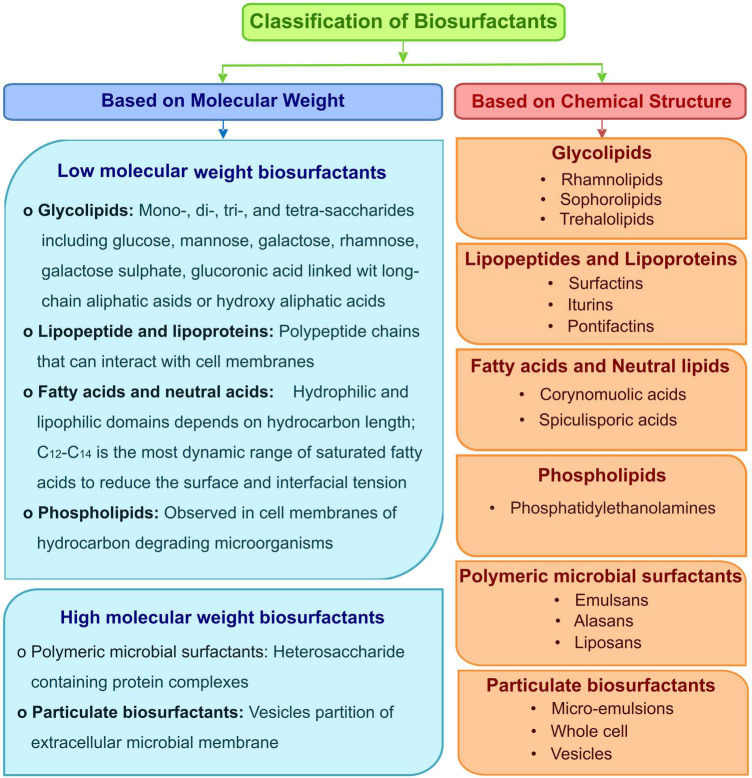
Classification of biosurfactants based on the chemical nature.

**FIGURE 2:**
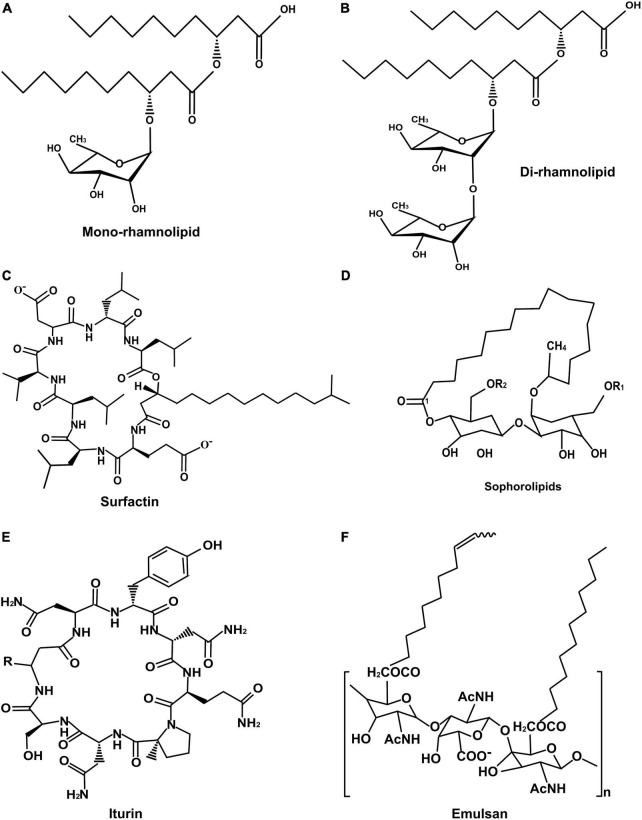
Structure of important biosurfactants: **(A)** Mono-rhamnolipid, **(B)** Di-rhamnolipid, **(C)** Surfactin, **(D)** Sophorolipid, **(E)** Iturin, and **(F)** Emulsan.

The nature of biosurfactants depends on the microbial origin and the nutrient availability, according to which they are classified into two categories based on their molecular weight and chemical composition (Figure 1). Based on the size, they are divided into two types, low molecular weight and high molecular weight biosurfactants. The low molecular weight biosurfactants can reduce the surface and interfacial tensions at the air and water interfaces. In contrast, high molecular weight biosurfactants are found effective in stabilizing the oil in water emulsions and are known as “bioemulsans.” They can work at low concentrations and have many substrate specificities, making them highly efficient emulsifiers. A few well-known biosurfactants’ chemical structures are given in Figure 2.

Furthermore, biosurfactants are classified based on their polarity as anionic or neutral compounds containing hydrophilic and hydrophobic domains. Carbohydrates, amino acids, phosphate groups, or other compounds are in the hydrophilic domain. In contrast, the hydrophobic domain is generally a long-chain fatty acid or derivative of fatty acids (Maneerat, 2005). Saranraj et al. (2022a) introduced some new biosurfactants like mannosylerythritol lipids (MELs), lichenysin, ituri, fengycin, viscosin, arthrofactin, amphisin, putisolvin, serrawettin, etc.

## Properties

**FIGURE 3:**
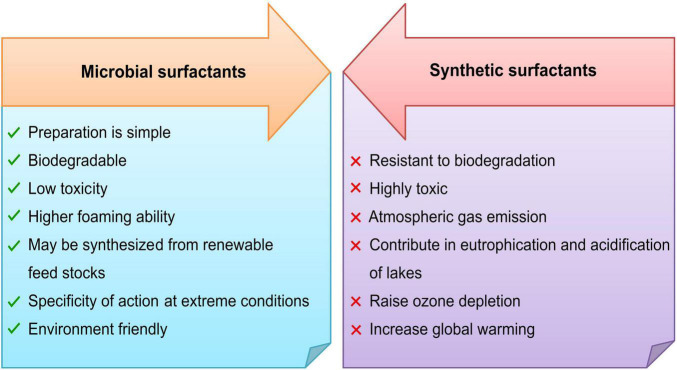
Microbial surfactants verses synthetic surfactants.

Synthetic surfactants are expensive and cause environmental problems because of toxicity and resistance to degradation. Microbial surfactants are the best alternative to synthetic surfactants as they show significant advantages over synthetic ones (Figure 3). The substantial properties of biosurfactants that makes them eligible to replace the synthetic surfactants are discussed here, which help evaluate their performance and selection of a potential microorganism.

### Surface and interfacial activity

**FIGURE 4:**
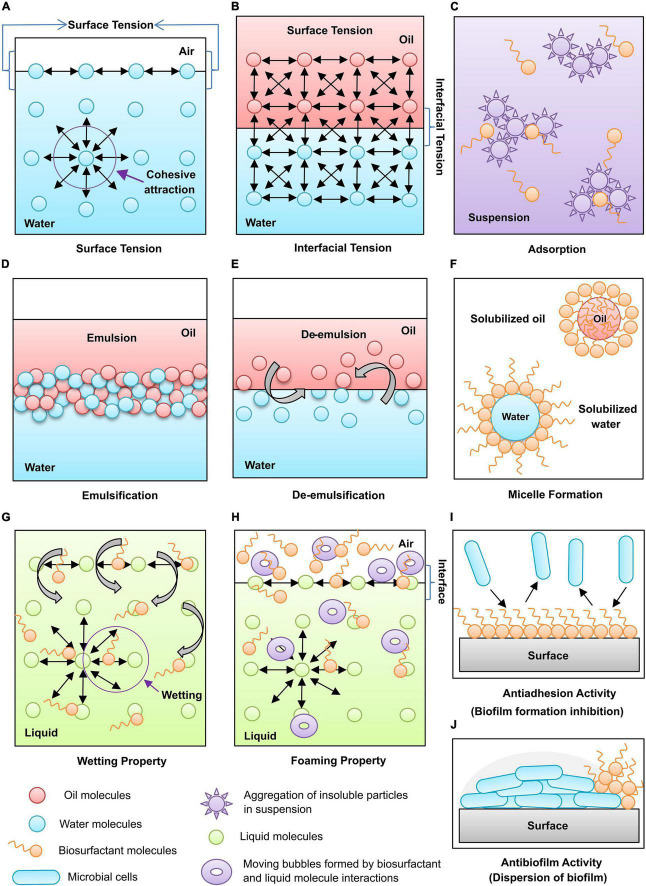
Functional properties of biosurfactants: **(A)** Surface tension, **(B)** Interfacial tension, **(C)** Adsorption, **(D)** Emulsification, **(E)** De-emulsification, **(F)** Micelle formation, **(G)** Wetting property, **(H)** Foaming property, **(I)** Antiadhesion activity, and **(J)** Antibiofilm activity.

Surface tension is created when the water droplet molecules are whispered together by a strong intermolecular and attractive, cohesive force on the surface (Figure 4A). Biosurfactants can reduce different solutions’ surface and interfacial tensions (Figure 4B) at very low concentrations because of their lower critical micelle concentrations (CMC).

### Emulsification

Biosurfactants can play a dual role, an emulsifier or a de-emulsifier. Emulsions are of two types: oil-in-water and water-in-oil emulsions. Generally, the emulsions prepared with two different phase solutions are not stable. The addition of biosurfactants allows dispersion of one liquid into another and helps two immiscible liquids to be mixed, which signifies micellular solubilization with large particles (Figure 4D).

### De-emulsification

The de-emulsification process breaks the emulsions by disrupting the stable surface between the internal and bulk phases (Figure 4E). This process helps to deal with the problems created by the natural emulsifying agents in oil recovery and production processes like corrosion of equipment used in the petroleum industries.

### Solubilization

A high concentration of biosurfactants will form micellar structures (Figure 4F), which encapsulate and transport the insoluble molecules at higher levels in the solution. They increase the solubility of water-insoluble substances in aqueous solutions or organic solvents. Biosurfactants are proved more efficient than synthetic surfactants in solubilizing the complex mixture of molecules into an aqueous solution.

### Wetting

A spreading and penetrating power of biosurfactants that reduces the surface tension of liquids by decreasing the attractive forces between similar particles and increasing affinity toward dissimilar surfaces is known as wetting ability. Biosurfactants can act as wetting agents by entering the pores rather than associating them with the surface tension (Figure 4G). Wetting agents is imperative when reconstructing dry compounds like powders, beads, or reagents in solid-phase devices.

### Foaming

Biosurfactants are concentrated on the gas-liquid interface to form fizzes through the liquid, forming foam formation (Figure 4H). The bubbling techniques study surface-active molecules’ foaming properties, e.g., surfactin, sodium dodecyl sulfate (SDS), and bovine serum albumin (BSA).

### Adsorption

Adsorption enables strong interactions between biosurfactants and hydrophobic substrates, which helps to enhance the recovery of biosurfactants from oil from rock or production media (Figure 4C). The biosurfactants’ adsorption property is the ability to act as an anti-adhesive agent (Figure 4I). Biosurfactants arbitrate the synthesis and stabilization of nanoparticles by adsorption which prevents aggregation and stabilization of nanoparticle formulations (Sadiq et al., 2022).

### Dispersion

Some biosurfactants are used as a dispersant to prevent the aggregation of insoluble particles with one another in the suspension. The reduction in cohesive attraction among similar particles leads to dispersion (Figure 4J). It desorbs the hydrophobic molecules from rock surfaces to enhance their mobility and recovery, which is helpful in oilfield applications. The dispersion also helps to inhibit or remove the biofilm formation of harmful microbes, hence biosurfactant are useful in making wound healing formulations.

### Flocculation

Flocculation is a process in which emulsion droplets stick together to form cluster-like structures called flocs. These flocs are not permanent and can be broken by mechanical action, thus restoring emulsions to their original form. Biosurfactants with flocculating ability have applications in environmental cleaning processes.

### Biodegradability

Being a microbial product, biosurfactants can easily be degraded in nature or in treatment plants without producing harmful end products. This most significant feature makes them a superior environment-friendly compound (Saranraj et al., 2022b).

### Low toxicity, biocompatibility, and digestibility

Biosurfactants are natural compounds with very low toxicity and can also be digested by humans, therefore widely used in the food and pharmaceutical industries. They also have righteous compatibility with many compounds used in cosmetics.

### Tolerance to extreme conditions

The biosurfactants produced by some extremophiles are popular because of their ability to resist extreme environmental factors like temperature, pH, and ionic strength. Ibrahim (2017) reported the rhamnolipids produced by *Ochrobactrum anthropic* HM-1 and *Citrobacter freundii* HM-2 with excellent stability at 50–100°C for 30 min, 2.0–12.0 pH, and 2–10% NaCl.

## Biosynthesis

Many researchers have studied biosynthetic pathways for the construction of biosurfactants. Being a biomolecule, each biosurfactant follows a different biosynthetic pathway as the nutritional and environmental conditions provided affect the microbial growth and its production, making them structurally diverse.

### Rhamnolipid biosynthesis

**FIGURE 5:**
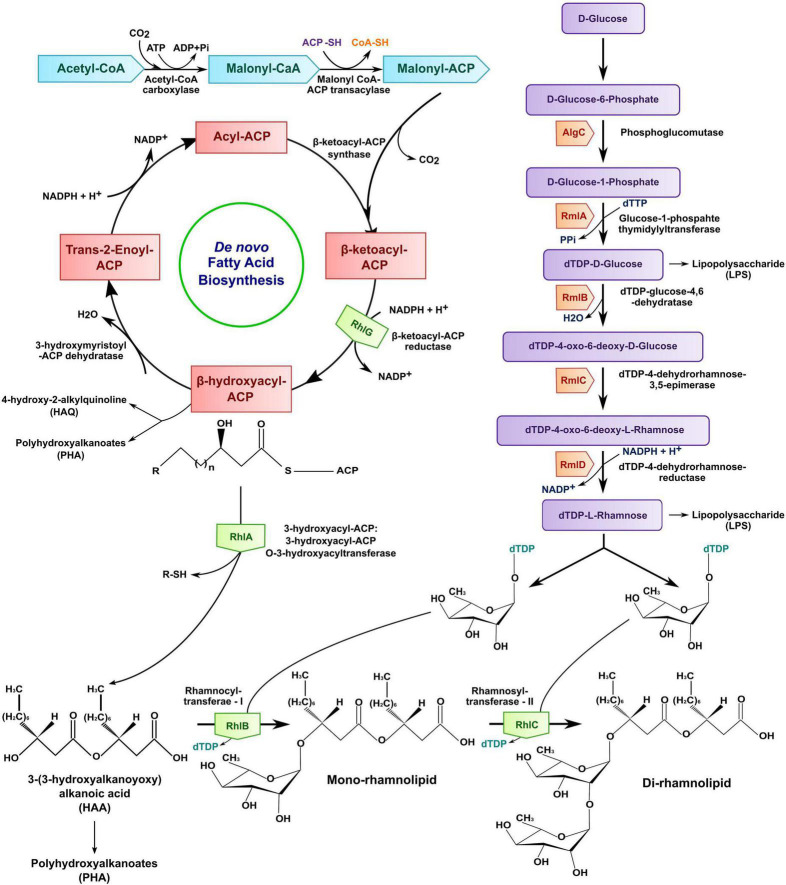
Biosynthesis of rhamnolipid by *Pseudomonas* spp.

The synthesis of fatty acid moieties for rhamnolipid differs from the general fatty acid biosynthesis at the ketoacyl reduction level (Kubicki et al., 2019). The *de novo* fatty acid biosynthesis supplies significant fatty acids to produce rhamnolipids by Pseudomonas aeruginosa as a model bacterium (Figure 5) for producing glycolipids. Rhamnose molecules are present in *P*. *aeruginosa* as a cell wall constituent in lipopolysaccharide (LPS). The rhamnose derives carbon from glycerol instead of acetate by condensing two carbon units formed by glycerol without splitting or rearranging their C–C bonds. Glycerol carbon provides all the carbons needed for rhamnolipid synthesis, whereas acetate can supply carbon for only β-hydroxydecanoic acid, an intermediate of β-oxidation.

Two glycosyltransferase units, i.e., rhamnosyltransferase I and rhamnosyltransferase II, primarily catalyze both mono- and di-rhamnolipids. The products of genes *rhlA* and *rhlB* organized by the bicistronic operon showed the sovereign activity of RhlA and RhlB proteins (Wittgens et al., 2017). The gene encodes for rhamnosyltransferase II, i.e., *rhlC* is localized at alternative chromosomal sites separately from *rhlA* and *rhlB* in *P*. *aeruginosa. rhlA* and *rhlC* genes are bound to the inner membrane, while *rhlB* is a membrane-bound gene. RhlA was studied to synthesize 3-(3-hydroxyalkanoyloxy) alkanoic acid (HAA) from the activated hydroxy fatty acid. In contrast, the glycosyltransferase RhlB catalyzes the condensation between dTDP-L-rhamnose (deoxy thymidine diphosphate L-rhamnose) and HAA to form mono-rhamnolipids. The RhlC involves di-rhamnolipid [L-rhamnose-L-rhamnose-3-(3-hydroxyalkanoyloxy) alkanoic acid] synthesis using mono-rhamnolipid as a substrate combined with dTDP-L-rhamnose. It shows sequence homology with rhamnosyltransferases linked in LPS synthesis (Pardhi et al., 2021b).

3-(3-Hydroxyalkanoyloxy) alkanoic acid already has surface-active properties and can be released in the cell’s environment as biosurfactants necessary for rhamnolipid production, but its function is unknown. RhlG enzyme is involved with rhamnolipid synthesis by draining the fatty acid precursors, and it also affects the polyhydroxyalkanoates (PHA) synthesis. HAA is a common compound involved in the origin of rhamnolipid and PHA synthesis, but PHA synthesis is not essential for rhamnolipids production. The RhlG provides the acyl carrier protein (ACP), a fatty acid precursor to synthesize the 4-hydroxy-2-alkylquinolines (HAQs) having QS-related *Pseudomonas* quinolone signal (PQS). The *rhlA*, *rhlB*, and *rhlC* genes are not only found in *P*. *aeruginosa* but are reported from other genera like *Burkholderia paseudomallei*, *Bacillus thailandensis*, and *Escherichia coli* as an essential protein for rhamnolipid synthesis (Varjani and Upasani, 2017).

Recent studies showed that the biosynthetic pathways involved with marine biosurfactants originated from non-marine bacteria (Kubicki et al., 2019). AlgC plays a central role in the biosynthetic pathway of dTDP-D-glucose, D-rhamnose, and dTDP-L-rhamnose. AlgC transforms D-glucose-6-phosphate to D-glucose-1-phosphate (precursor of dTDP-D-glucose and dTDP-L-rhamnose), which is used to produce LPS and exopolysaccharide alginate. RmlA, RmlB, RmlC, and RmlD are enzymes of the rmlABCD operon, catalyzing the dTDP-L-rhamnose pathway in *P*. *aeruginosa*.

### Surfactin biosynthesis

**FIGURE 6:**
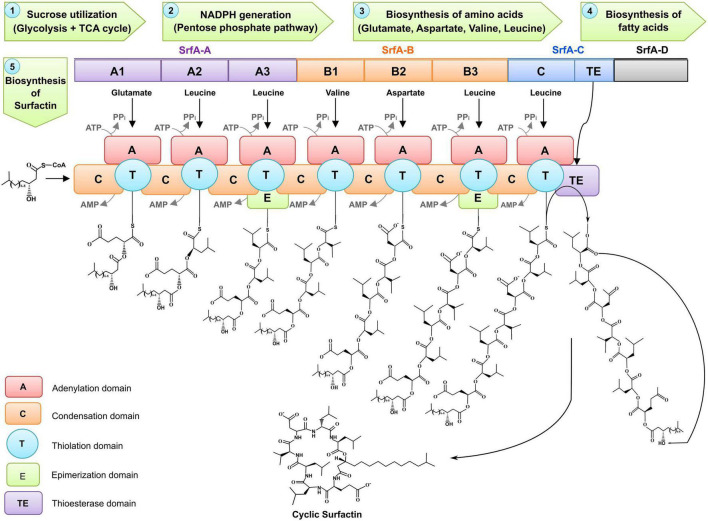
Biosynthesis of surfactin by *Bacillus* spp.

The general biosynthetic pathway of surfactin produced by *Bacillus subtilis* is shown in Figure 6. A special character called non-ribosomal peptide synthetases (NRPS) catalyzed by multi-enzymatic thiotemplates are assembled modularly to synthesize surfactin, a lipopeptide biosurfactant. This multi-modular enzymatic assembly carries acyl chain initiation, elongation, and termination, catalyzed through protein molecules. The NRPS catalyzes reactions like incorporating lipids, lactonization, or epimerization. Each module contains different domains and helps incorporate and change one specific amino acid in the peptide chain. A prototypic module contains three domains, i.e., condensation, adenylation, and thiolation domain/peptidyl carrier protein (PCP) domain. The condensation domain catalyzes direct condensation of the thioesterified intermediates in the growing chain. An adenylation domain selects the amino acid for the respective module and releases the pyrophosphate by catalyzing the aminoacyl adenosine formation from adenosine triphosphate (ATP) and cognate amino acid. The thiolation domain supports the covalent bonding of activated amino acids, and the 4′-phosphopantetheine prosthetic group exists on the PCP through a thioester linkage (Shaligram and Singhal, 2010).

The epimerization domains usually help transform L- to D-amino acids. It shows that the composition of non-ribosomal peptides contains amino acids except proteinogenic ones. The operon *srfA* (25 kb) determines that NRPS comprises three multi-functional proteins encoded by *srfA-A srfA-B*, and *srfA-C.* The proteins SrfA-A (402 kDa), SrfA-B (401 kDa), SrfA-C (144 kDa), and a small subunit SrfA-D (40 kDa) are important for the initiation reactions of surfactin (Shaligram and Singhal, 2010). SrfA-A and SrfA-B are three-modular proteins; SrfA-C is a mono-modular with a thioesterase domain, and SrfA-D is a subunit (Figure 6; Kubicki et al., 2019).

A starter molecule, 3-hydroxy fatty acid, classically known as 3-hydroxy-13-methyl-myristic acid, was recognized by the first module’s condensation domain, containing seven amino acids (L-glutamate, L-leucine, D-leucine, L-valine, L-aspartate, D-leucine, and L-leucine) are successively added through seven modules. The thioesterase (TE) domain of termination module SrfA-C catalyzes the product and lactonization of the depsipeptide after the entire acyl chain is synthesized. These TE domains are chain-terminating protein moieties (25–30 kDa) generally found in the fatty acid biosynthesis. Some TE domains are reported as hydrolases and some for carrying regio- and stereo-specific reactions, while TE domains of SrfA-C are noted with a prominent intramolecular cyclization feature. An acyl-O-TE intermediate is engaged for intramolecular detention by a nucleophilic group of the acyl chain instead of undergoing hydrolysis (Kohli et al., 2001; Tanovic et al., 2008). Ali et al. (2022a) has discussed the influence of quorum sensing and CRISPRi technology on surfactin.

## Microorganisms

Some microorganisms can use various substrates considered potentially harmful to other non-biosurfactant-producing microbes and produce structurally diverse biosurfactants. The composition and yield of the biosurfactant produced exclusively depend upon the sites from where the microorganisms are isolated, their genetic makeup, physiological conditions, and the various nutrients utilized by the organisms. Oil-contaminated sites like crude oil contaminated localities, petrochemical industrial waste, tannery effluents, used edible oils, and oil reservoirs are the major spots for the collection of samples for isolation of potential biosurfactant producers. Moreover, extremophiles are also reported from marine environments to produce extensively stable biosurfactants.

The genera *Pseudomonas* and *Bacillus* are very well explored for biosurfactant production contributing approximately 50–60% of the reported bacteria (Table 1). However, several fungi like *Candida* spp. and *Pseudozyma* spp. are also recognized as the principal biosurfactants producers. The bacterial producers are discovering each type of biosurfactant, while fungi are reported with a maximum production of glycolipids such as sophorolipids and mannosylerythritol lipid (Table 2).

**TABLE 1:** Bacterial strains producing biosurfactants.

Biosurfactants	Bacteria	Carbon sources	References
***Pseudomonas* sp.**			
**Lipopeptide**	*Pseudomonas guguanensis* D30	Mineral oil	Pardhi et al., 2021a
	*Pseudomonas putida* MTCC 2467	Sucrose	Kanna et al., 2014
**Rhamnolipid**	*Pseudomonas aeruginosa* OG1	Chicken feather	Ozdal et al., 2017
	*Pseudomonas fluorescens* 1895	Olive oil/*n*-hexadecane	Abouseoud et al., 2007
	*Pseudomonas aeruginosa* ATCC 10145	Waste frying oil	Wadekar et al., 2012
	*Pseudomonas aeruginosa* AT10	Soybean oil refinery waste	Abalos et al., 2001
**Polymeric**	*Pseudomonas stutzeri*	Diesel	Joshi and Shekhawat, 2014
***Bacillus* sp.**			
**Iturin**	*Bacillus subtilis*	Glucose/rapeseed oil, crude oil	Bayoumi et al., 2010
**Phospholipids**	*Bacillus sphaericus* EN3, *Bacillus azotoformans* EN16	Glucose/diesel/crude oil	Adamu et al., 2015
**Lipopeptide**	*Bacillus subtilis* LB5a	Cassava wastewater	Nitschke et al., 2006
	*Bacillus* sp.	Dextrose	López-prieto et al., 2019
	*Bacillus subtilis* CN2	Coal tar creosote	Bezza and Chirwa, 2015
	*Bacillus licheniformis* Y-1	Olive oil/diesel/crude oil/kerosene	Liu et al., 2016
	*Bacillus subtilis*	Soybean, sweet potato residues	Wang et al., 2008
**Surfactin**	*Bacillus subtilis*	Crude oil	Pereira et al., 2013
**Other genera**			
**Trehalose-2,3,4,2′-tetraester**	*Bordetella hinzii*-DAFI	Sucrose/molasses, crude oil	Bayoumi et al., 2010
**Phospholipid**	*Klebsiella pneumoniae IVN51*	Dextrose	Astuti et al., 2019
**Glycolipid**	*Ochrobactrum anthropi* HM-1, *Citrobacter freundii* HM-2	Waste frying oil	Ibrahim, 2017
	*Pseudoxanthomonas* sp. G3	Heavy oil	Astuti et al., 2019
	*Nocardia otitidiscaviarum*	Crude oil	Vyas and Dave, 2011
**Polymeric**	*Serratia marcescens* UCP 1549	Corn waste oil	Araújo et al., 2019
	*Stenotrophomonas maltophilia* UCP 1601	Soybean/corn/diesel	Nogueira et al., 2020
**Lipopeptide**	*Virgibacillus salarius*	Waste frying oil	Elazzazy et al., 2015
	*Stenotrophomonas* sp. B-2	Crude oil	Gargouri et al., 2017
	*Aeromonas salmonicida*	Gasoline	Kamal et al., 2015
**Exopolysaccharide**	*Gordonia polyisoprenivorans* CCT 7137	Sugarcane molasses	Fusconi et al., 2010
**Rhamnolipid**	*Burkholderia thailandensis*	Glycerol	Dubeau et al., 2009
	*Pseudoxanthomonas* sp.	Hexadecane	Nayak et al., 2009
	*Ralstonia pickettii* SRS, *Alcaligenes piechaudii* SRS	Crude oil	Płaza et al., 2008
**Bioemulsan**	*Gordonia* sp. BS29	Aliphatic hydrocarbons	Franzetti et al., 2009

**TABLE 2:** Fungal strains producing biosurfactants.

Biosurfactants	Fungi	Carbon sources	References
**Filamentous fungi**			
**Glycolipid**	*Penicillium citrinum*	Olive oil	Camargo-de-Morais et al., 2003
**Uzmaq**	*Aspergillus flavus* AF612	Glucose	Ishaq et al., 2015
**Lipopeptide**	*Penicillium chrysogenum* SNP5	Wheat bran and grease waste	Gautam et al., 2014
	*Fusarium* sp. BS-8	Sucrose and yeast extract	Qazi et al., 2013
**Fatty acids**	*Fusarium oxysporum*	Crude oil	Santhappan and Pandian, 2017
**Complex Carbohydrate/protein/lipid**	*Cunninghamella echinulate* UCP	Soybean waste oil and corn steep liquor	Silva et al., 2014
**Yeasts**			
**Microbial lipids**	*Cryptococcus curvatus*	Acetate	Gong et al., 2015
**Sophorolipids**	*Pichia anomala* PY1	Soybean oil	Thaniyavarn et al., 2008
	*Starmerella bombicola* ATCC 22214	Sweetwater	Wadekar et al., 2012
	*Candida bombicola* ATCC 22214	Turkish corn oil and honey	Pekin et al., 2005
	*Candida lipolytica* IA 1055	Babassu oil	Vance-Harrop et al., 2003
**Lipopeptide**	*Candida lipolytica*	Groundnut oil	Rufino et al., 2007
**Mannosylerythritol lipids**	*Candida antarctica*	*n*-Alkanes	Kitamoto et al., 2001
	*Ustilago scitaminea* NBRC 32730	Sugarcane juice	Morita et al., 2009
	*Pseudozyma tsukubaensis*, *Pseudozyma fusifornata, Pseudozyma parantarctica*	Soybean oil	Morita et al., 2006
	*Pseudozyma aphidis*	*n*-Hexane	Rau et al., 2005
	*Kluyveromyces marxianus* FII 510700	Lactose	Lukondeh et al., 2003
	*Kurtzmanomyces* sp. I-11	Soybean oil	Kakugawa et al., 2002
	*Pseudozyma siamensis* CBS 9960	Sunflower oil	Morita et al., 2008
**Glycolipid**	*Wickerhamomyces anomalus* CCMA 0358	Olive oil/soybean oil/glucose	Souza et al., 2017
	*Candida antarctica*, *Candida apicola*	Oil refinery waste	Bednarski et al., 2004

*Brevibacterium casei* MSA19, *Streptomyces* spp. MAB36, *Bacillus circulans*, *Aspergillus ustus* MSF3, and *Nocardiopsis alba* MSA10 are a few marine microbes producing biosurfactants used in the medical field as they exhibit antimicrobial, anti-adhesive, and anti-biofilm activities against human pathogens (Gudiña et al., 2016). Besides natural strains, some mutant or recombinant strains like *Pseudomonas aeruginosa* 59C7, *Bacillus licheniformis* KGL11, *Acinetobacter calcoaceticus* RAG-1, *Gordonia amarae* gave 2–4 times more yield than the native strains (Mukherjee et al., 2006).

## Production

Microorganisms utilize a wide range of complex organic substrates to get carbon and energy by converting them into simpler forms through fermentation. They produce significant products like ethanol, amino acids, vitamins, polysaccharides, etc. Biosurfactants are one of the secondary metabolites produced during such fermentation processes. Submerged and solid-state fermentations are used for biosurfactant production based on the microorganism’s nature.

### Substrates

The choice of a suitable substrate is critical for commercially and economically effective biosurfactant manufacturing. Researchers have explored inexpensive resources to replace the costlier substrates, such as agro-industrial wastes, vegetable oil mill effluents (coconut, canola, olive, grape seed, palm, rapeseed, sunflower, soybean oil), dairy and sugar industry byproducts (buttermilk, whey, molasses), starch industry extract and wastes (corn, potatoes, tapioca, wheat) (Saranraj et al., 2022c). Using these substrates will reduce production costs while also helping conserve the environment. The low-cost carbon sources are utilized to increase the biosurfactant yield (Tables 1, 2).

### Submerged fermentation

Submerged production processes are ideal for biosurfactant-producing bacteria and yeasts as they require water for optimum growth. Biosurfactants are extracellular compounds released by bacteria in the fermentation broth, making them simple to purify. However, some valuable compounds may have been known to leach out of the liquid portion during recovery, which is a disadvantage of submerged fermentation (SmF). Many researchers have designed the mineral salt medium and studied the submerged biosurfactant production using the shake flask method (Pardhi et al., 2020). De Rienzo et al. (2016) carried out a rhamnolipid production in a 10 L laboratory-scale bioreactor using *Burkholderia thailandensis* E264 and *Pseudomonas aeruginosa* ATCC 9027. *Candida bombicola* and *Pseudomonas aeruginosa* were reported with 34 and 20 g/L sophorolipids in 50 L bioreactor, respectively (Shah et al., 2007; Zhu et al., 2007).

### Solid state fermentation

Solid state fermentation (SSF) generally uses solid materials such as molasses, wheat bran, cassava dregs, rice husk, cassava bagasse, coffee husk, banana peel, tapioca peel, etc., as a substrate are usually low-cost, carbon and protein-rich renewable wastes. Successful solid-state fermentations are reported for biosurfactant production by *Aspergillus fumigatus*, *Phialemonium* spp., and *Pleurotus ostreatus* using rice husk with defatted rice bran, soy oil or diesel oil, and sunflower seed oil, respectively (Martins et al., 2006; Velioğlu and Öztürk Ürek, 2015). In addition, some bacterial strains like *Serratia rubidaea* SNAU02, *Brevibacterium aureum* MSA13, and *Bacillus pumilus* UFPEDA 448 showed more rhamnolipids and lipopeptides production using SSF than SmF (Kiran G. et al., 2010; Slivinski et al., 2012; Nalini and Parthasarathi, 2014).

## Recovery and purification

The economic recovery and downstream processes account for almost 60% of total production costs, will ensure the commercial viability of a bioprocess. Biosurfactants’ physicochemical features, such as surface or micelle forming activity, make them easier to recover than other secondary metabolites. The most often reported methods for biosurfactant recovery are listed in Table 3.

**TABLE 3:** Downstream processes for biosurfactant recovery.

Recovery method	Separation mechanism	Significance	References
**Batch**			
**Acid precipitation**	Acid/base changes the solutions pH to biosurfactants isoelectric point (pH = pI), which makes them insoluble molecules	Inexpensive, suitable for recovery of crude biosurfactants	Sen and Swaminathan, 2005
**Crystallization**	The filtered broth treated with suitable solutions to get relatively insoluble crystals of biosurfactants in precipitated form	Used in initial recovery and final purification of compounds	Stanburry et al., 2016
**Organic solvent extraction**	Biosurfactants contain hydrophobic ends which solubilize them in organic solvents	Reusable, useful in crude biosurfactant recovery, inexpensive	Kuyukina et al., 2001
**Ammonium sulfate precipitation**	Salting out	Use to extract polymeric biosurfactants	Stanburry et al., 2016
**Continuous**			
**Centrifugation**	Central force precipitates the insoluble biosurfactants	Inexpensive, reusable, convenient for crude biosurfactant recovery	Nitschke et al., 2006
**Foam fractionation**	Surface activity makes participation of biosurfactants into foam	High purity level	Noah et al., 2002
**Adsorption**	Adsorptive materials adsorbed the biosurfactants and desorbed using organic solvents	One step recovery, high level of purity, fast, reusable	Dubey et al., 2005
**Membrane ultrafiltration**	Biosurfactants form micelles above their CMC which get trapped by polymeric membranes	Fast, one step recovery, high level of purity	Sen and Swaminathan, 2005
**Tangential flow filtration**	A membrane allows the liquid to pass through, separates the biosurfactant	Efficient separation is independent of cell and media densities and no filter aid needed	Stanburry et al., 2016
**Ion exchange chromatography**	Charged biosurfactants are attached to the ion exchange resins and eluted using suitable buffers	High level of purity, fast, reusable	Abd-al-hussan et al., 2016

Biosurfactants are extracted mainly by organic solvents but most of them are toxic; hence researchers have replaced them with low toxic and cheap solvents that reduce the recovery expenses. A single downstream process is not sufficient to recover and purify the biosurfactant. Hence, multi-step recovery strategies with a series of purification and concentration steps are used, allowing for better quality recovered products at different stages. Crude biosurfactants can be obtained for environmental cleanup at a low cost with only a few early recovery processes.

## Characterization

Various chromatographic and spectrophotometric methods are widely used for biosurfactant characterization individually or in combination, depending on the type of biosurfactant. The structural characterization of the biosurfactants will help to figure out their applications in different fields.

The phospholipids, rhamnolipids, and lipopeptides were separated by thin layer chromatography (TLC) using chloroform:methanol:water solvent system (Pekin et al., 2005; Daverey and Pakshirajan, 2009; Nwaguma et al., 2016). High-performance liquid chromatography (HPLC) is generally used to separate and identify the lipopeptide-type biosurfactants. For glycolipids, the HPLC device must be coupled with an evaporative light scattering detector (ELSD) or mass spectrometry (MS). It was observed that HPLC coupled with other devices like ultra-HPLC-MS are faster than the qualitative HPLC. Recently Bartal et al. (2018) identified surfactin isomers from *Bacillus subtilis* SZMC 6179J using HPLC-ESI-MS (electrospray ion-mass spectrometry).

Fourier Transform-Infrared Spectroscopy (FT-IR) analysis through classical KBr disk was used for lipopeptides produced by *Bacillus* spp. and *Virgibacillus salaries* (Elazzazy et al., 2015; López-prieto et al., 2019). In recent years, a new FT-IR approach has been introduced, i.e., attenuated total reflectance (ATR) crystal accessory which give rapid and more effective results. Reports of surfactin analysis through ATR confirmed it as a successful improved technique of FT-IR (Bezza and Chirwa, 2015; Pardhi et al., 2021a). Daverey and Pakshirajan (2009) identified the chemical configurations of sophorolipid and trehalose lipid through NMR. Mass spectrophotometry (MS) is generally coupled with other techniques for better performance like gas chromatography-MS (GC-MS), electrospray ion-MS (ESI-MS), secondary ion-MS (SIMS), liquid chromatography-ESI-MS (LC-ESI-MS), ultra-high-performance liquid-high-resolution-MS (UHPLC-HRMS), and matrix-assisted laser desorption/ionization-time of flight-MS (MALDI TOF-MS). The newly discovered biosurfactants, lichenysin-A, and aneurinifactin are purified and characterized by MALDI TOF-MS (Joshi et al., 2016; Balan et al., 2017).

## Patents and worldwide production of biosurfactants

The demand for biosurfactants is progressively growing as the most desirable green surface-active product to replace the synthetic one. But the high cost of production prevents them from becoming the most considerable product in their field; therefore, researchers are emphasizing an ideal biosurfactant producing strains, alternative low-cost substrates, and minimal bioreactor process. To achieve these approaches, researchers have studied many biosurfactants and published the patents with their exclusive properties (Table 4).

**TABLE 4:** Patents of biosurfactant production and applications.

Patent no.	Patent title	References
US 20190029250A1	Preventing and destroying citrus greening and citrus canker using rhamnolipid	Desanto, 2019
US 20160030322A1	Application of surfactin in cosmetic products	Lu et al., 2016
WO 2017029175A1	Improved lactam solubility	Price, 2016
US 20130296461B2	Aqueous coatings and paints incorporating one or more antimicrobial biosurfactants and methods for using same	Sadasivan, 2015
US 20140080771B2	Method for treating rhinitis and sinusitis by rhamnolipids	Leighton, 2013
EP 2410039A1	Rhamnolipids with improved cleaning	Unilever Plc., 2012
WO 20120255918A1	Use of rhamnolipids in the water treatment industry	DeSanto and Keer, 2012
US 8183198B2	Rhamnolipid-based formulations	Desanto, 2012
WO 2011109200A9	The use of rhamnolipids as a drug of choice in the case of nuclear disasters in the treatment of the combination radiation injuries and illnesses in humans and animals	Piljac, 2012
US 20150336999A1	Process for the production of sophorose starting from sophorolipids	Jourdier and Chhabban, 2012
US 20110306569A1	Rhamnolipid biosurfactant from *Pseudomonas aeruginosa* strain NY3 and methods of use	Yin et al., 2011
WO 2013037818A3	Beverages containing glycolipid preservatives	Schloesser et al., 2011
US 7968499B2	Rhamnolipid compositions and related methods of use	Gandhi and Skebba, 2011
US 8685942B2	Sophorolipid analog compositions	Gross and Schofield, 2011
US 9351485B2	Use of sophorolipids and derivatives thereof in combination with pesticides as adjuvant/additive for plant protection and the industrial non-crop field	Giessler-Blank et al., 2009
WP 2008/001921	Dermatological anti-wrinkle agent	Eiko and Toshi, 2008
KR 20090117081	Conditioning shampoo composition containing biosurfactant	Seok, 2008
US 8648055B2	Virucidal properties and various forms of sophorolipids	Gross et al., 2004
WO 2006069175A3	Antifungal properties of various forms of sophorolipids	Gross and Shah, 2004
US 20040152613A1	Detergent compositions – glycolipids	Develter et al., 2003

Recently, Allied Market Research stated that the global chemical surfactants market size was valued at 41.3 billion USD in 2019 and is projected to reach 58.5 billion USD by 2027, registering a compound annual growth rate (CAGR) of 5.3% from 2020 to 2027 (Dixit et al., 2020). While according to the survey by Global Market Insight, the biosurfactants market size exceeded 1.5 billion USD in 2019 and is expected to grow at over 5.5% CAGR between 2020 and 2026 (Ahuja and Singh, 2020). Increasing emphasis on replacing petrochemical-based surfactants owing to high toxicity, low sustainability, and shelf-life should drive the product demand. The financial requirements of large-scale biosurfactant production are high, yet some companies manufacture biosurfactants globally (Table 5) to fulfill the public demand. Among all the biosurfactants, the rhamnolipids has the highest market share and is expected to grow over 5% CAGR in the future, especially in the Asia-Pacific region, owing to high consumption from countries like India, Japan, and China (Ahuja and Singh, 2020). After rhamnolipids, sophorolipids are the most selling products in the cosmetic sector (Table 5).

**TABLE 5:** Worldwide manufacturers of biosurfactants.

Location	Biosurfactant	Company	Application field
**India**	Rhamnolipid/Surfactin	Altinbio Scientific Pvt. Ltd.	Personal care, cleanser, medical, agriculture, wastewater treatment
	Unknown	Geocon Products	Shampoo, cosmetics
		Akshay Intensive Marketing	Detergent preparations and cosmetics
**United Kingdom**	Rhamnolipid	Unilever and Evonik	Household cleaning products
	Rhamnolipid/lipopeptide	TeeGene Biotech	Pharmaceuticals, antimicrobial and anti-cancer components, cosmetics
**South Korea**	Sophorolipid	MG Intobio Co. Ltd.	Beauty products, bath soaps
**United States**	Rhamnolipid	AGAE Technologies LLC	Pharmaceutical, cosmetics, enhanced oil recovery, personal care, bioremediation (*in situ* and *ex situ*)
		NatSurFact Laboratories	Personal care, cleaning
		Jeneil Biosurfactant Co. LLC	Cleaning products, enhanced oil recovery
		Paradigm Biomedical Inc.	Pharmaceuticals
		Rhamnolipid Companies, Inc.	Agriculture, pharmaceuticals, cosmetics, enhanced oil recovery, bioremediation, food products
	Sophorolipid	Synthezyme LLC	Cleaning products, cosmetics, food products, fungicides, crude oil emulsification
**Germany**	Glycolipid	Fraunhofer IGB	Pharmaceuticals, washing liquids
	Rhamnolipid/Sophorolipid	Henkel	Laundry, glass cleaning, beauty products
**France**	Sophorolipid	Groupe Soliance	Cosmetics
**Japan**	Sophorolipid	Kaneka Co.	Cosmetics, toiletry products
		Saraya Co. Ltd.	Cleaning, sanitation products
		Allied Carbon Solutions Ltd.	Agricultural products
	Methyl-ester sulfonate	Lion Corporation	Detergent’s formulations, cleaning products
**Canada**	Rhamnolipid	EcoChem Organics Company	Hydrocarbon diffusive agent
**Belgium**	Sophorolipid	Ecover Belgium	Cleaning products, cosmetics, bioremediation

## Applications of biosurfactants

**FIGURE 7:**
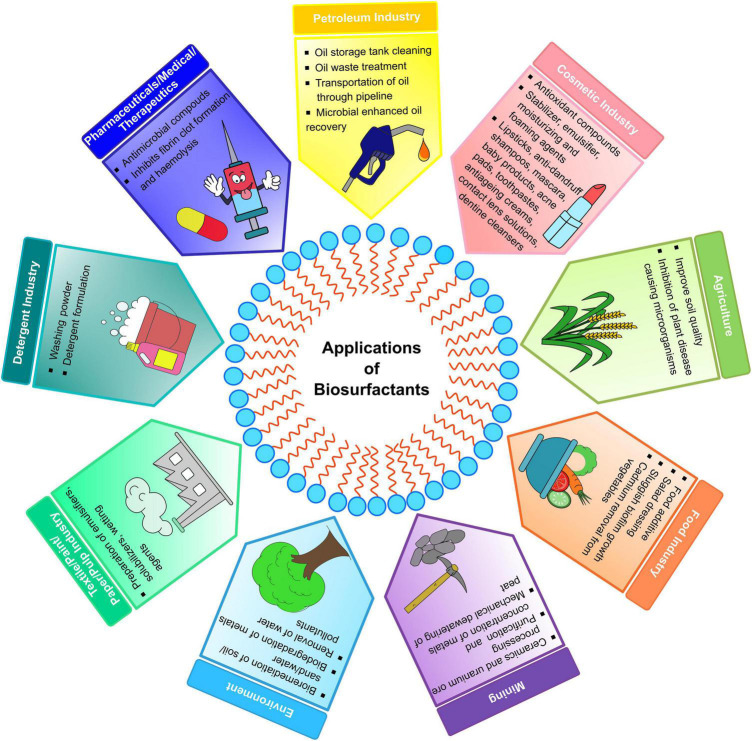
An overview of potential applications of biosurfactants in different fields.

Biosurfactants are significant compounds having the potential to replace synthetic surfactants. They have many applications in industrial sectors like petroleum, organic chemicals, pharmaceuticals, cosmetics, foods and beverages, bioremediation, petrochemicals, biological control, etc. (Figure 7). The potential biosurfactants and their applications are reported in Table 6.

**TABLE 6:** Potential biosurfactants and their applications.

Biosurfactant		Applications	References
**Glycolipids**	**Rhamnolipid**	Hydrocarbon degradation and dispersion enhancement	Whang et al., 2008
		Antimicrobial activity	Abalos et al., 2001
		Emulsification of hydrocarbons and vegetable oils	Dubeau et al., 2009
		MEOR and dye solubilization	Hultberg et al., 2008; Christova et al., 2013
		Removal of metals from soil	Hormann et al., 2010
	**Sophorolipid**	Recovery of hydrocarbons from dregs and muds; removal of heavy metals from sediments	Whang et al., 2008
		Reducing and stabilizing agent	Parekh and Pandit, 2012
		Degradation of diesel oil	Chandran and Das, 2010
		Anti-cancer activity	Jing et al., 2007
	**Trehalose lipid/trehalolipid**	Antiviral activity and inhibition of phospholipase A2	Zaragoza et al., 2013
		Hemolytic and antibacterial activity	Zaragoza et al., 2010
		Oil spill cleanup operations by hydrocarbon solubilization	Peng et al., 2007
	**Trehalose tetraester**	Bioremediation of oil-contaminated sites	Tuleva et al., 2009
	**Xylolipid**	Surface and antibacterial activity	Joshi-Navare et al., 2014
	**Mannosylerythritol lipid**	Washing detergent capacity	Morita et al., 2008
		Antimicrobial, immunological, and neurological properties	Shibahara et al., 2000
**Lipopeptide**	**Crude cyclic lipopeptide**	Laundry detergent additives	Mukherjee, 2007
		Antioxidant activities, phenanthrene solubilization and re-mobilization of hydrocarbons from contaminated soil	Gargouri et al., 2017
		Gasoline degradation	Kamal et al., 2015
		Antimicrobial, anti-adhesive, antitumor activities	Cao et al., 2009
		Biocontrol agent and fertilizer synergist	Wang et al., 2008
	**Aneurinifactin**	Crude oil removal from contaminated sand	Balan et al., 2017
	**Ponctifactin**	Antimicrobial and anti-biofilm activity; MEOR	Balan et al., 2016
	**Surfactin**	MEOR	Pereira et al., 2013
		Remediation of petroleum contaminated soil	Liu et al., 2016
	**Lichenysin-A**	Recovery of residual oil from sandstone	Joshi et al., 2016
	**Serrawettin W2**	Chemorepellent	Pradel et al., 2007
	**Friulimicin B**	Antibacterial property	Schneider et al., 2009
	**Iturin**	Antimicrobial activity	Ahimou et al., 2001
**Phospholipids**	**Phosphatidylethanolamine**	Hydrocarbon emulsification	Nwaguma et al., 2016
**Polymeric**	**Alasan**	Hydrocarbon stabilization and emulsification	Toren et al., 2002

### Petroleum industry

Biosurfactants augment the removal and biodegradation of oil through mobilization, de-emulsification, solubilization, or emulsification. Rhamnolipids and surfactins showed better petroleum removal capacity than the synthetic surfactants from soil. The glycolipids from *Ochrobactrum anthropic* HM-1, *Citrobacter freundii* HM-2, and *Pseudoxanthomonas* spp. G3 efficiently recovered 70%, 67%, and 20% of residual oil from the sand-packed column (Ibrahim, 2017). In addition, Jain et al. (2012) recovered >90% lubricant oil from sandy soil using 1% (w/v) biosurfactant. Alike bacteria, *Fusarium* spp. BS-8 (JQ860113) was also reported with 46% enhanced oil recovery (Qazi et al., 2013). Rhamnolipid (0.4 mg/mL) was reported to remove 90% Mb, 30% Ni, and 70% Vd. In comparison, lipopeptide (17.34 mg/mL) removed 44.5% carbon from the harmful spent hydrodesulfurization (HDS) catalyst produced by petroleum refineries (Alsaqer et al., 2018). The cleaning and maintenance of oil storage containers are often problematic, as hazardous compounds used for cleaning generate a massive volume of harmful wastes. An oil sludge fraction deposited on the walls or bottom of the storage tanks is incredibly viscous semisolid particles and difficult to remove using conventional pumping. Oil-contaminated vessels were cleaned within 15 min using a biosurfactant of *P*. *aeruginosa* SH 29 (Diab and Din, 2013).

### Environment

Biosurfactants are used in environmental protection for oil spill control and detoxifying oil-contaminated industrial effluents and soils. Their ability to stabilize oil/water emulsions and increase the hydrocarbon solubility enhances biodegradation and removal of hydrocarbon from the soil (Shah et al., 2022). An environment-friendly surfactin was reported with 100% biodegradation of activated sludge within 4 days (Fei et al., 2019). Rhamnolipids had efficiently removed Ni and Cd from soils (80–100%) and field samples (20–80%) (Mulligan and Wang, 2004). The crude oil (89%) was desorbed through lipopeptide (Al-dhabi and Esmail, 2020) and efficiently gas-oil was removed (86.7%) from soil by rhamnolipid (Gonzini et al., 2010). Obayori et al. (2009) reported 95.29% and 92.34% degradation of diesel and crude oil using biosurfactant. An emulsion of rhamnolipid-silica nanoparticles efficiently worked as a dispersant to remediate the crude oil-seawater system (Pi et al., 2015). For a sustainable environment, the most prominent field for the application of biosurfactants is bioremediation.

### Agriculture

Biosurfactants are used for various purposes in agriculture, such as improving soil quality, removal of common water-soluble pollutants, helping to eliminate plant pathogens, supporting valuable plant-microbe interactions, pesticide preparations, etc. The rhamnolipid removed pentachlorophenol (PCP) from sand-soil (60%) and sandy-silt soils (61%) (Mulligan and Eftekhari, 2003). A biosurfactant reported with 72% degradation of anthracite related to Fe-stimulation within 48 days (Santos et al., 2008). Bee et al. (2019) observed efficient antifungal activity of rhamnolipid and surfactin against *Fusarium oxysporum f*. spp. *ricini*. A lipopeptide allegedly inhibited the anthracnose-causing pathogen *Colletotrichum gloeosporioides* in papaya leaves (Kim et al., 2010). A surfactin was used to treat the *Rhizoctonia solani* infected maize crop which led the production of defense enzymes (Ali et al., 2022b). Such properties make biosurfactants useful in phytopathogenic control. The biosurfactant from *Serratia marcescens* UCP 1549 was reported with 125% stimulation of cabbage seed germination (Araújo et al., 2019). A glycolipid significantly stimulated the growth promoting factors of *Capsicum annuum L*. (Ravinder et al., 2022).

### Detergent industry

Now-a-days, public awareness is rising for the environmental risks linked with synthetic surfactants. Hence, a demand for eco-friendly biosurfactants which can substitute the laundry detergent is stimulated for soaps, shampoos, and washing liquids preparations. The biosurfactant forms micelles to remove the oily stains from the desired material by attracting their hydrophilic moieties. The detergent mixture of surfactin and subtilisin A efficiently removed immobilized rubisco stain from hydrophilic (75%) and hydrophobic (80%) surfaces (Onaizi et al., 2009). A rhamnolipid (0.01%) competently removed the marker stains from the whiteboard (Turbekar et al., 2014). The biosurfactant produced by *Klebsiella* spp. RJ-03 was reported to remove up to 80% lubricant oil from cotton cloth (Jain et al., 2012). Similarly, rhamnolipid, lipopeptide, and glycolipid removed 61.43% sunflower oil, 75% motor oil, 81% tea stains, and 86% burned engine oil from cotton fabric (Bafghi and Fazaelipoor, 2012; Bouassida et al., 2018).

### Medical industry

The toxicity of biosurfactants is exerted on the permeability of cell membranes in a manner similar to that of detergents. Biosurfactants have biological properties such as antibacterial, anti-adhesive, anticancer, anti-mycoplasma, and hemolytic, making them a viable compound in the medical and cosmetic sectors. The rhamnolipids have shown antimicrobial activity against *Aspergillus niger*, *Gliocladium virens*, *Chaetomium globosum*, *Penicillium chrysogenum*, *Aureobasidium pullulans*, *Botrytis cinerea*, *Rhizoctonia solani*, *Penicillium chrysogenum*, *Candida albicums, Bacillus pumilus*, *Micrococcus luteus*, and *Sarcina lutea* (Abalos et al., 2001; El-Sheshtawy and Doheim, 2014). Lunasan, a new biosurfactant, has demonstrated antimicrobial activity against *Streptococcus oralis* (68%), *Staphylococcus epidermidis* (57.6%), *Candida albicans* (57%) and also exhibited anti-adhesive effect against *Streptococcus agalactiae* (100%), *Streptococcus sanguis* (100%), *Pseudomonas aeruginosa* (92%) (Luna et al., 2011). Thanomsub et al. (2007) reported rhamnolipid A and B having anti-proliferative activity against human breast cancer cell line and insect cell line C6/36 with a minimum inhibitory concentration of 6.25 μg/mL and 50 μg/mL, respectively. A water soluble polysaccharide kefiran produced by *Lactobacillus kefiranofaciens* ATCC 43761 showed anticancer activity with 193.89 μg/mL of IC_50_ against breast cancer (MCF-7) cells (Dailin et al., 2020). These properties make biosurfactants a suitable applicant for biomedical preparations.

### Cosmetic industry

Cosmetic applications are one of the extraordinary parts of multifunctional biosurfactants. The applications depend on their excellent surface properties, including emulsification, detergency, solubilization, dispersion, wetting, and foaming effects. They also showed antioxidant activity, anti-irritating effects, and compatibility with skin with better moisturizing properties (Patel et al., 2022). Rhamnolipids, sophorolipids, and mannosylerythritol lipids (MELs) exhibit skin compatibility, low skin-irritation, and moisturizing properties, replacing the petrochemical-based surfactants applied in top cosmetic preparations like anti-wrinkle and anti-aging products (Table 4). MELs are introduced in the cosmetic field for exclusive liquid-crystal-forming and moisturizing assets and are mainly used in preparations preventing skin roughness. A sodium dodecyl sulfate (SDS)-damaged human skin cells showed 77.1% viability and self-assembling property after penetration of di-acylated MEL-B, which formed lyotropic liquid crystals to moisturize the skin (Morita et al., 2011). Concaix (2003) reported sophorolipids as stimulators of skin fibroblast metabolism, which helps in restoring, protecting, and repairing skin. They also reduce the subcutaneous fat overload by stimulating leptin synthesis in adipocytes, allowing cellulite treatment (Pellecier and André, 2004). MEL-A (0.5%) and MEA-B (0.5%) are studied for increasing the tensile strength of damaged hairs up to 122 gf/p and 119.4 gf/p; hence can be used for damaged hair treatment (Morita et al., 2010).

### Food industry

Biosurfactants generally play a role in food formulating ingredients as fat stabilizers, food emulsifiers, and anti-adhesive agents. It is also used to control the agglomeration of fat globules, stabilize aerated systems, improve the texture and shelf-life of starch-containing products, modify the rheological properties of wheat dough, and improve the consistency and texture of fat-based products. Biosurfactants can decrease the adhesion of pathogenic organisms to solid surfaces or infection sites, hence used to protect the food products (Zaman et al., 2022). A biosurfactant extracted from *Lactobacillus paracasei* spp. *paracasei* A20 showed anti-adhesive activity against *L. reuteri* (77.6–78.8%), *L*. *casei* (56.5–63.8%), *Streptomyces sanguis* 12 (72.9%), *S*. *mutans* HG985 (31.4%), *Staphylococcus aureus* (76.8%), *S*. *epidermidis* (72.9%), *S*. *agalactiae* (66.6%), *Pseudomonas aeruginosa* (21.2%), *E*. *coli* (11.8%) (Gudiña et al., 2010). Long-term consumption of heavy metal contaminated vegetables may cause numerous human health hazards. A glycolipid was reported with 59% biofilm inhibition, 73% Cd removal from garlic, and antimicrobial activity against *E*. *coli* (Anjum et al., 2016). The biosurfactants increased the emulsion stability of fruit salad dressing from 51.4 to 62.8% (Sridhar et al., 2015). The muffins treated with lipopeptide were observed to reduce hardness and stickiness and showed improved softness (Kiran G. S. et al., 2010). A new glycolipid, diacyl mannosyl erythritol, showed an ice-packing factor of 35% for 8 h, thus helpful in improving ice slurry’s storage ability (Kitamoto et al., 2001).

### Miscellaneous applications

Besides these, biosurfactants are commercially used in pulp, paper, paint, plastic, leather, and textile industries, along with ceramics and uranium ore processing. This is because the biosurfactants have de-resinification and pulp washing, defoaming, color smoothing, antistatic agent, pigment dispersion, coating, latex stabilization, retard sedimentation, emulsification, and wetting capability. The polymeric biosurfactant has shown potential as a wood adhesive material (Pervaiz and Sain, 2010). A biosurfactant producing *Cobetia marina* is patented as an additive of paint formulation for submersible surfaces (Dinamarca-Tapia et al., 2012). Rhamnolipid (Raza et al., 2014) and saponin (Leighs et al., 2018) are reported for scouring cotton fibers and wools, respectively. The biosurfactant-producing *Meyerozyma guilliermondii* and *Acidithiobacillus* spp. co-inoculated to solubilize the toxic metals like Zn (76.5%), Ni (59.8%), Cu (22%), Cr (9.8%), Cd (9.8%), and Pb (7.1%) from sewage sludge in 10 days, hence suitable for bioleaching (Camargo et al., 2018).

## Conclusion

Biosurfactants possess the fundamental physico-chemical properties like surface tension reduction, micelle formation, emulsification and adsorption as like chemical surfactants but low toxicity and biodegradability give them edge over the synthetic one. Apart from known producers like *Bacillus* and *Pseudomonas*, many other genera like *Burkholderia*, *Serratia*, *Klebsiella*, *Pseudozyma*, and *Fusarium* were reported for biosurfactants. Rhamnolipids are the most widely used biosurfactants followed by sophorolipids in industries. A number of new biosurfactants with diverse applications are also introduced, namely aneurinifactin, ponctifactin, lichenysin-A, and friulimicin-B. Biosurfactants are in high demand as a prospective product in industries like petroleum, healthcare, cosmetics, detergents, agriculture, medicine, the environment, and food due to their beneficial characteristics. The potential of biosurfactants to replace synthetic surfactants and dominate the global market is hindered by their high manufacturing costs, despite the fact that they are a green surface-active product with steadily rising demand. Abundant opportunities exist to explore novel microbial strains that produce novel biosurfactants using inexpensive alternative substrates with minimal bioreactor process. The biodegradable microbial surfactants will be highlighted as one of nature’s most promising products for the environmental preservation and healthy future generations.

## Author contributions

KR contributed to the conceptualization and supervision. DP contributed to the methodology and writing – original draft. RP, VR, and RJ contributed to the formal analysis. KR, PP, and WA contributed to the writing – review and editing. WA contributed to the fund acquisition. All authors contributed to the article and approved the submitted version.
