# Supplementary figures and images for: Microbial surfactants: A journey from fundamentals to recent advances
Source: Front Microbiol. 2022 Aug 4;13:982603. doi: 10.3389/fmicb.2022.982603 (PMC9386247; doi:10.3389/fmicb.2022.982603)

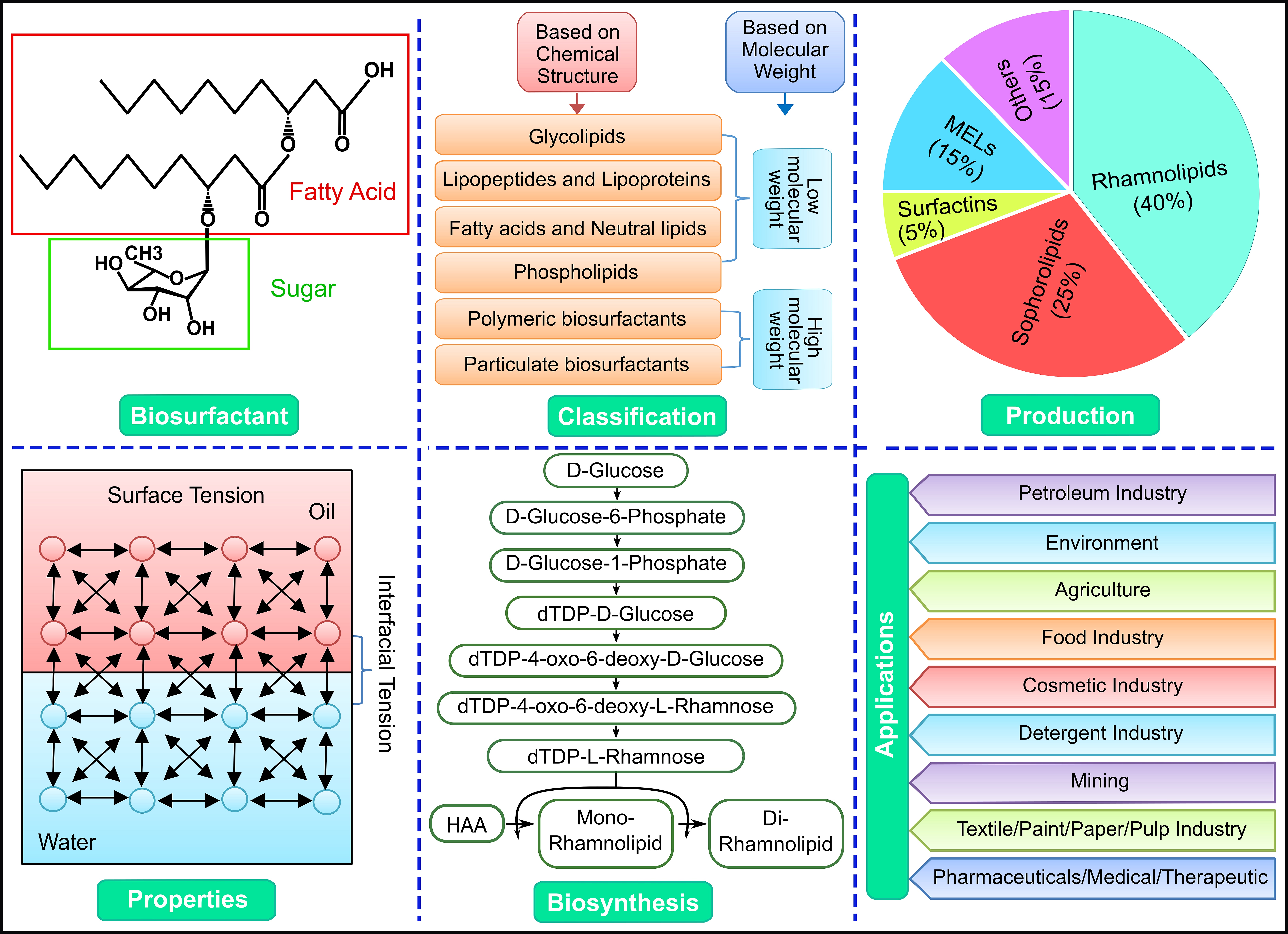

Supplement: Supplementary file 1 [file Image_1.JPEG]
